# Analyses of fracture line distribution in intra-articular distal radius fractures

**DOI:** 10.1007/s11547-019-01025-9

**Published:** 2019-03-22

**Authors:** Xin Zhang, Yinqi Zhang, Jian Fan, Feng Yuan, Qian Tang, Cory J. Xian

**Affiliations:** 1 Department of Orthopedics, Tongji Hospital, School of Medicine, Tongji University, 389 Xincun Road, Shanghai, 200065 China; 2 School of Pharmacy and Medical Sciences and University of South Australia Cancer Research Institute, University of South Australia, Adelaide, SA 5001 Australia

**Keywords:** Intra-articular distal radius fracture, Fracture line, Fracture mapping, Heat mapping

## Abstract

**Purpose:**

To assess the association between the fracture line distribution and the location of comminution in intra-articular distal radius fractures by building fracture mapping.

**Patients/methods:**

Forty cases with intra-articular fractures of distal radius were enrolled in the current prospective clinical study. Fracture lines and comminution zones were identified by reduced three-dimensional computed tomography reconstructions and then graphically superimposed onto a standard template to create two-dimensional fracture maps, followed by the conversion into heating maps. Based on qualitative descriptive fracture mapping analyses, the patterns of intra-articular distal radius fractures were determined.

**Results:**

It was observed that the highest fracture line intensity was located as an inverted “T” shape zone in the dorsal aspect of the joint with high incidence of fractures and the prominently intense color in heat mapping. The keystone projected area, the radial styloid process and the metacarpal radial side articular surface were found to be the least involved parts of the fracture. According to the mapping of the number and distribution of fracture lines, a new classification method for intra-articular fractures of the distal radius was redefined. Different surgical approaches and internal fixation techniques were proposed for different types. In this paper, we retrospectively compared the preoperative X-ray findings between different types. Based on the preoperative X-ray prediction, the distal intra-articular radius fractures were classified, so as to develop effective surgical strategies. In this study, a new surgical approach was attempted, but due to the lack of evidence-based evidence, long-term postoperative complications and hand function should be further evaluated.

**Conclusion:**

This study redefines a new method for the classification of intra-articular fractures of the distal radius, which allows doctors to have a clearer understanding of the characteristics of distal radius fractures. Moreover, the application value in fracture diagnosis is more significant, and the best surgical approach is selected for different types.

## Introduction

Distal radius fracture (DRF) is one of the most common types of fractures presented in emergency clinics, among which intra-articular fractures account for approximately 25% of all DRFs [1]. This high incidence trend is continuing to increase with the aging populations and increased numbers of high-energy injuries (e.g., motor vehicle accidents). Although the surgical management for DRFs is variable, the principle is to restore the bone anatomic structures and to recover the function of the wrist joint after rigid internal fixation as soon as possible. Recently, with the development of volar locking palmar plates, which have been demonstrated to have a low-profile design, a capacity of neutralizing the load across the fracture sites and the non-requirement of good bone quality, volar locking plate fixation has been widely used in the treatment of distal radius intra-articular fractures. However, it is still relatively complicated and challenging for surgeons to restore the articular surfaces and to fix important articular fracture fragments to achieve early rehabilitation. In particular, due to the unexposed joint capsule through the volar locking approach, the location and dependence of internal fixation for fractures of the distal radius articular surface is principally relied on X-ray examinations and surgeons’ experience, requiring precise and effective methods. Therefore, it has become a strong interest for surgeons to know how to achieve the articular surface fracture fixation after restoring the articular surface and whether to add other screws when volar locking plate distal screws are used to fix important fracture fragments, especially for the elderly patients with osteoporosis [2]. Recently wrist arthroscopy may be an effective method to observe the fracture line of distal fractures but not popular because of technique and expense [3].

The current study aimed to explore the distribution characteristics of the fracture lines in DRFs by building fracture maps reconstructed using axial computed tomography (CT) scan images of patients with intra-articular fractures of distal radius. As fracture mapping can contribute to the better understanding and accurate evaluation on the fracture patterns of DRFs (e.g., location and frequency of the injury of the articular surface), through fracture mapping, surgeons hence could further anticipate the fracture patterns through superimposing fracture lines, zones of comminution and articular involvement, which provide novel mechanistic insights and develop therapeutic strategies of DRFs [4].

## Materials and methods

### Patients

Consecutive image sampling was conducted for the patients with distal radial fractures at Department of Orthopedics of Tongji Hospital (Tongji University, Shanghai, China) between July 2014 and January 2016 by using the International Classification of Diseases, Ninth Revision, Clinical Modification (ICD-9-CM) codes. The inclusion criteria were listed as below: (1) intra-articular fracture of distal radius; (2) patient aged 18 years or older; and (3) preoperative CT scans with adequate quality (e.g., slice thickness below 1.5 mm). Patients with open and/or old fractures were excluded. In total, 40 of 265 patients with distal radial fractures were screened for entry into the current study.

### Fracture mapping

**Fig. 1:**
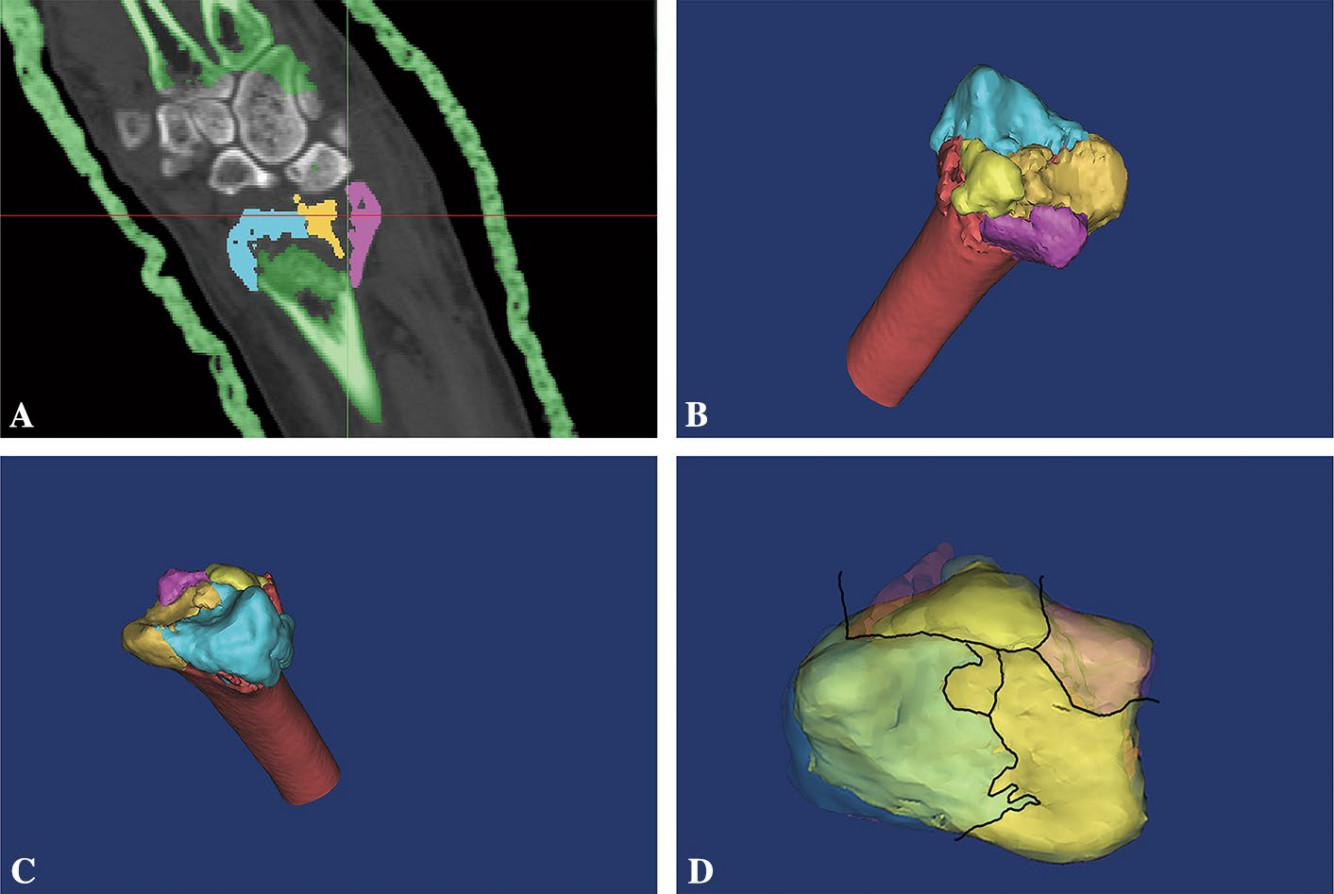
Schematic illustration of fracture mapping. **a** Free bone fragments marked with different colors; **b** A 3D reconstructed model; **c** A 3D image manually reset; **d** A 3D image with the fracture lines drawn

**Fig. 2:**
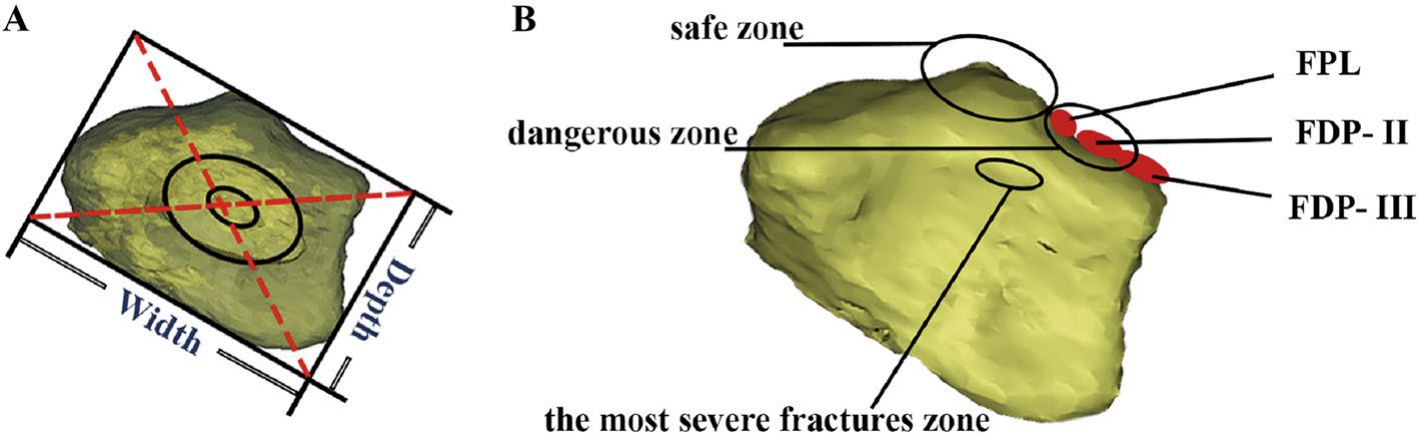
Illustration of the standard morphological analysis for the distal radius articular surface and the related parameters. **a** In the process of 3D reconstruction, the backbone of radius was in translucent mode and the position of the backbone was then adjusted to form the ellipse through overlapping the margin of the bony cortex. By adjusting the parameters in the diagram (width and depth), the center of the ellipse was adjusted at the centroid of the articular surface (i.e., the intersection of dashed red lines) to form a standard articular surface. **b** Illustration of the articular view and some important anatomy landmarks such as flexor pollicis longus (FPL), the dangerous zone (FPL would be threatened in this zone during the volar plate implanting) and the safe zone. FDP-II = index finger; FDP-III = middle finger

Three-dimensional computed tomography (Q3DCT) analyses were performed to visualize the distributions and locations of fracture lines. Firstly, the images of intra-articular fractures of the distal radius were obtained by Q3DCT techniques for two-dimensional (2D) fracture mapping. The selected CT images were then reloaded into software MIMICS 17.0 (MIMICS, Boston, MA), and bone structures were marked manually in axial, sagittal and coronal planes to create 3D polygon mesh reconstructions. In MIMICS program, all displaced fragments were restored to their original locations and marked with different colors. The anatomical landmarks were matched to ensure proper rotation and alignment. The fracture lines therefore could be distinguished based on the free bone fragments of different colors (Fig. 1). The basis of all image overlays was used to establish distal radius articular surface as previously described [5], in which the practical values of width and depth of articular surface of distal radius are standardized to 29.0 mm and 20.8 mm, respectively (Fig. 2). All the articular images were subsequently superimposed by software Photoshop CC (version 2015.5; Adobe Systems Incorporated, San Jose, CA) to build a frequency diagram as determined by the density of fracture lines.

### Heat mapping and data analysis

As a mean to obtain a more intuitive understanding of the 2D fracture mapping, the initial diagrams were also converted into fracture heat maps by using Photoshop CC (Adobe Systems Incorporated). The heat maps were created based on the fracture lines and represented the relative frequency of fracture lines that have been graphically displayed as colors.

The analysis of the fracture maps was descriptive. Heat mapping was conducted to visually summarize the fracture lines and compare for differences. In the heat mapping, the distribution frequency of the fracture on the articular surface is distinguished by the change in appearance of the intensity.

## Results

There were 23 (57.5%) female and 17 (42.5%) male cases for intra-articular fractures of the distal radius eligible in the current study. The average age was 61.1 years, ranging from 25 to 85 years. Fracture etiology included falls (18 cases) and traffic accidents (22 cases). According to the Association for Osteosynthesis/Association for the Study of Internal Fixation (AO/ASIF) fracture classification system, there were 3 cases (7.5%) of type B1, 2 cases (5%) of type B2, 2 cases (5%) of type B3, 11 cases (27.5%) of type C1, 8 cases (20%) of type C2 and 14 cases (35%) of type C3 intra-articular fracture of distal radius.

**Fig. 3:**
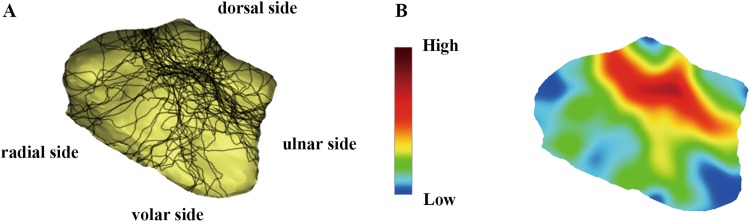
The distribution and heat mapping of the fracture lines in the comminution zone of the intra-articular distal radius fractures. **a** The fracture lines were distributed on the distal radius surface. **b** The intensity of the color represents the frequency of the fracture occurrence, and fracture line intensity is illustrated by heat mapping analyses

In the current qualitative descriptive research, it was observed that the highest fracture line intensity was located as an inverted “T” shape zone in the dorsal aspect of the joint, which has the prominently intense color in heat mapping and divides the distal radius into three parts (Fig. 3). In particularly, most of the fracture lines were observed to be vertically distributed. On the volar aspect of the joint, the fracture lines were shown to be less distributed and dispersed, which are accompanied with the lower color intensity in heat mapping. In addition, the keystone projected area, the radial styloid process and the metacarpal radial side articular surface were found to be the least involved parts in the intra-articular fractures of the distal radius (Fig. 3).

## Discussion

Distal radius fracture (DRF) is one of the most common type of fractures. As the functions of the wrist joint mostly depend on the integrity and axial direction of the distal radius, precise reconstruction of the articular surface and rigid fixation of main fracture fragments are the key to reduce the risk of traumatic osteoarthritis and achieve satisfactory function recovery. According to the traditional surgical principles, the dorsal or volar procedure should be performed for the treatment of homologous displaced fractures. Recently, with the development in advanced fixation techniques, more than 90% of DRFs could be effectively fixed through a volar locking plate. In this fixation technique, to be able to insert the distal locking screws into the proper locations and to the accurate depth is well known to be the foundation to achieve adequate fixed capacity and the compression of debris. However, the proper treatment of intra-articular fracture of distal radius requires great understanding of the fracture pattern and optimal preoperative planning for surgeons; and thus, there is an urgent requirement of the surgeons in predictably defining the fracture patterns in the articular surface of distal radius before operation. In particular, the fracture locations and vectors are vital for the surgical tactic and even possibly for the development of peri-articular plates. Although fracture mapping techniques have obtained the substantial advancements and have been widely used in clinical practice to further understand the injury patterns [5].

**Fig. 4:**
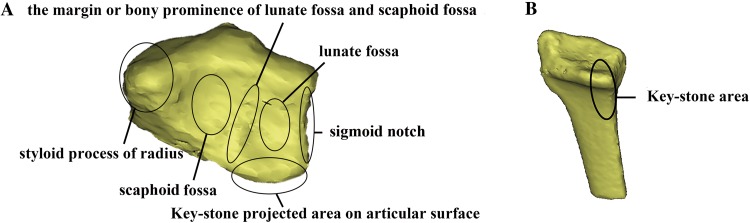
Schematic illustration of the distal radius surface. Some important anatomical landmarks are illustrated as below. **a** Lunate fossa and scaphoid fossa: two independent stress distribution areas in the radial articular surface, which could form the radiocarpal joint with the lunar and scaphoid; Sigmoid notch: an important anatomical structure of the radius, which forms the distal radioulnar joint with ulnar head and serves as the pivot of forearm pronation and post-pronation. **b** The keystone area: the intermediate column (IC) in the three-column biomechanical construction and the main conduction area of the energy load which is often used as a key point for screw placement

In the current study, intra-articular distal radius fractures were mapped by 3D reconstruction of articular surface with the data transferred from preoperative CT examination, which is currently widely used in assessing the distal radius fractures [6, 7]. Through the 3D studies for osteoarticular surface, the distribution of fracture lines could be analyzed stereoscopically based on anatomical landmarks. The 3D images were subsequently projected into 2D images (Fig. 4). Thus, articular surface details such as the ankle, fossa and protrusion of the articular surface were clearly displayed.

**Fig. 5:**
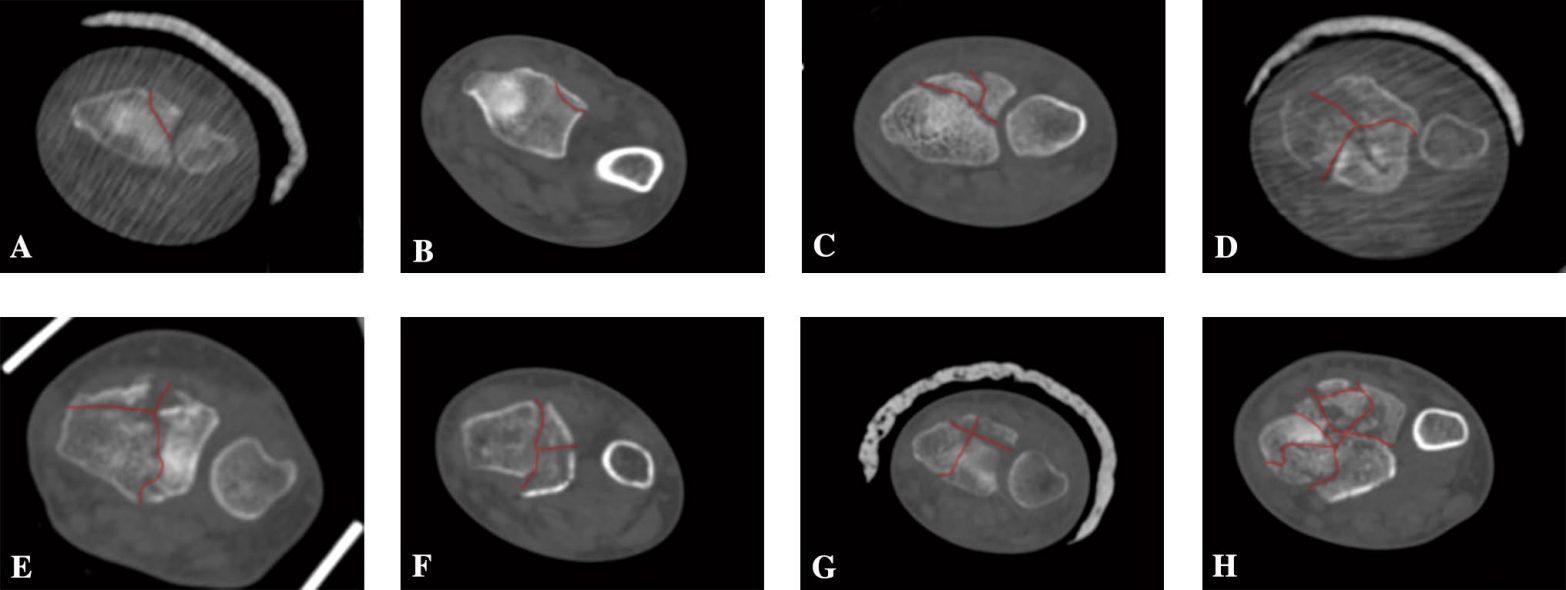
Showing the typical manifestations of our classification of distal radius intra-articular fractures in CT images. The fracture line marked with red line. **a, b** the fracture of a single fracture line; **(C)** a typical “┴” type of fracture; **d** a typical “┬” type of fracture; **e** a typical “┤” type of fracture; **f** a typical “├” type of fracture; **g** a typical “┼” type of fracture; **h** the fracture of multiple fracture lines

In addition, by analyzing fracture line distribution, the location of the fragments could be inferred, and the area of the metaphyseal collapse (effective bone loss) companied with potential instability also could be estimated. The classification of distal radial intra-articular fractures is defined based on the number and distribution of fracture lines. Since the targeted treatment for the fracture type with a single fracture line is relatively simple, it will not be further discussed in the current manuscript. On the other hand, we will focus on fracture type with the common distribution of double fracture lines. Based on our preliminary studies, the fracture line distribution could be divided into five categories: “┴,”“┬,”“┤,”“├” and“┼” (Fig. 5). In the case of multiple fracture lines, it can refer to the “十” type, in which there are at least 3 fracture lines. Two of the fracture lines traverse the articular surface and intersect.

As the most common type, “┴” type is observed to be with more bone loss and less lunate and scaphoid fossa surface. According to the mapping analyses, the main dorsal fragment was shown to be 6.9 mm in length on average (ranging from 3.8 to 10.1 mm) from the dorsal edge of the articular surface. In heat mapping, the fracture lines in the dorsal aspect of the intra-articular DRFs were found to mainly commence at the basilar part of the radial styloid process, horizontally running across the dorsal margin of the lunate fossa and scaphoid fossa, and terminating at the middle part of the sigmoid notch (Fig. 4). In addition, consistent with previous studies [8], it was observed that those sagittal fracture lines intensively start at the junction between safe zone and dangerous zone and then concentrate in the bony prominence between lunate fossa and scaphoid fossa (Fig. 3). Dorsal approach is thus recommended in surgery.

In addition, there was also acceptable bone loss found in “┬” type, which could be treated by volar approach surgery, so that bone grafting may not be performed intraoperatively. The fracture lines at the lunate fossa or scaphoid fossa were characterized to be intensively distributed at the edge of the fossa and directly through the center of the fossa sparsely. Previous studies have shown that the reconstruction of the articular surface of the lunate fossa and scaphoid fossa is critical to facilitate the recovery of the wrist function and to reduce postoperative complications [9].

Since “├” type fractures involved sigmoid notch, aside from reconstruction of the distal radioulnar joint surface and exploring the damage of surrounding soft tissue (TFCC), the volar approach cold also be achieved. It was found that the sigmoid notch, which has the high incidences of the comminuted fractures, is the most severely affected region with fractures. According to the fracture mapping analyses, the fracture lines were observed to be widely and randomly distributed in this area, accompanied with the free bone fragments. As the triangular fibrocartilage complex (TFCC) was considered as the major ligamentous stabilizer of the distal radioulnar joint [10], more attention should be paid for restoring the stability of TFCC, which is dependent on the distribution of the fracture lines at the sigmoid notch, during the fracture reduction and fixation.

For “┤” type fractures, the focus of reduction is on the styloid process of the radius. Volar approach could achieve strong internal fixation. “┼” type is the most serious intra-articular fracture with serious bone loss, requiring simultaneous approach of volar and dorsal as well as bone graft during the operation.

**Fig. 6:**
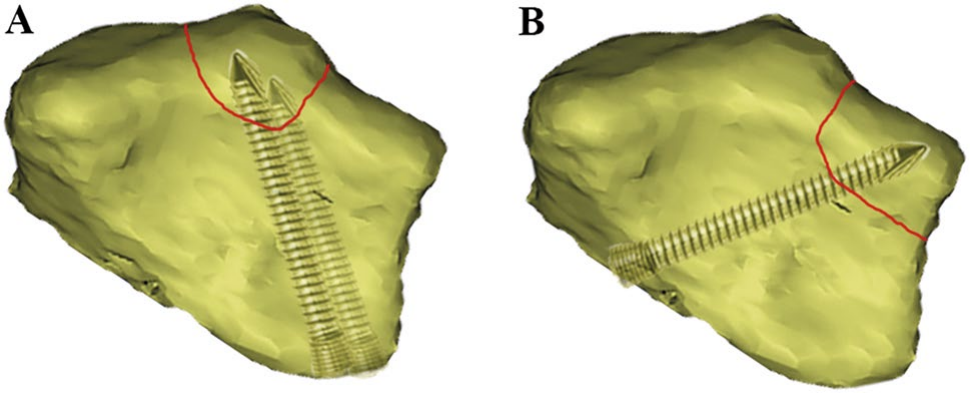
Schematic illustration of the projection of screws on the articular surface in the intra-articular distal radius fracture. **a** The standard screw position in the mainstream volar locking plate. **b** The assumption of the screw being twisted from the metacarpal radial side to dorsoulnar side. The red line represents the fracture line

In the current study, the so-called keystone area was found to be intact in 75% of the intra-articular fracture cases, while 17.5% cases were found to have simple fractures in this area (Fig. 4). In addition, the keystone area also remained intact in 64.3% type C3 fracture cases. These findings indicated that the keystone area is not an isolated articular surface of the distal radius but an important area with dense trabecular bone structure in the dorsal aspect of the distal radius, strongly supporting the rationality and safety of the keystone area screwing. Apart from the keystone area, the radial styloid process was shown to be the area with the lowest fracture rate, which suggests it could be served as another screw inserting point. Therefore, two screws could be inserted from the styloid process of radial to dorsoulnar to make the dorsoulnar comminution fixed steadily (Fig. 6). However, due to the lack of a precise intraoperative instrument to assess the positions (orientation and depth) of the screws, it still relies on the surgeon’s experience and skill level to reach the proper position and decrease iatrogenic complications (e.g., the extensor tendon problems) in the clinic [11, 12].

**Fig. 7:**
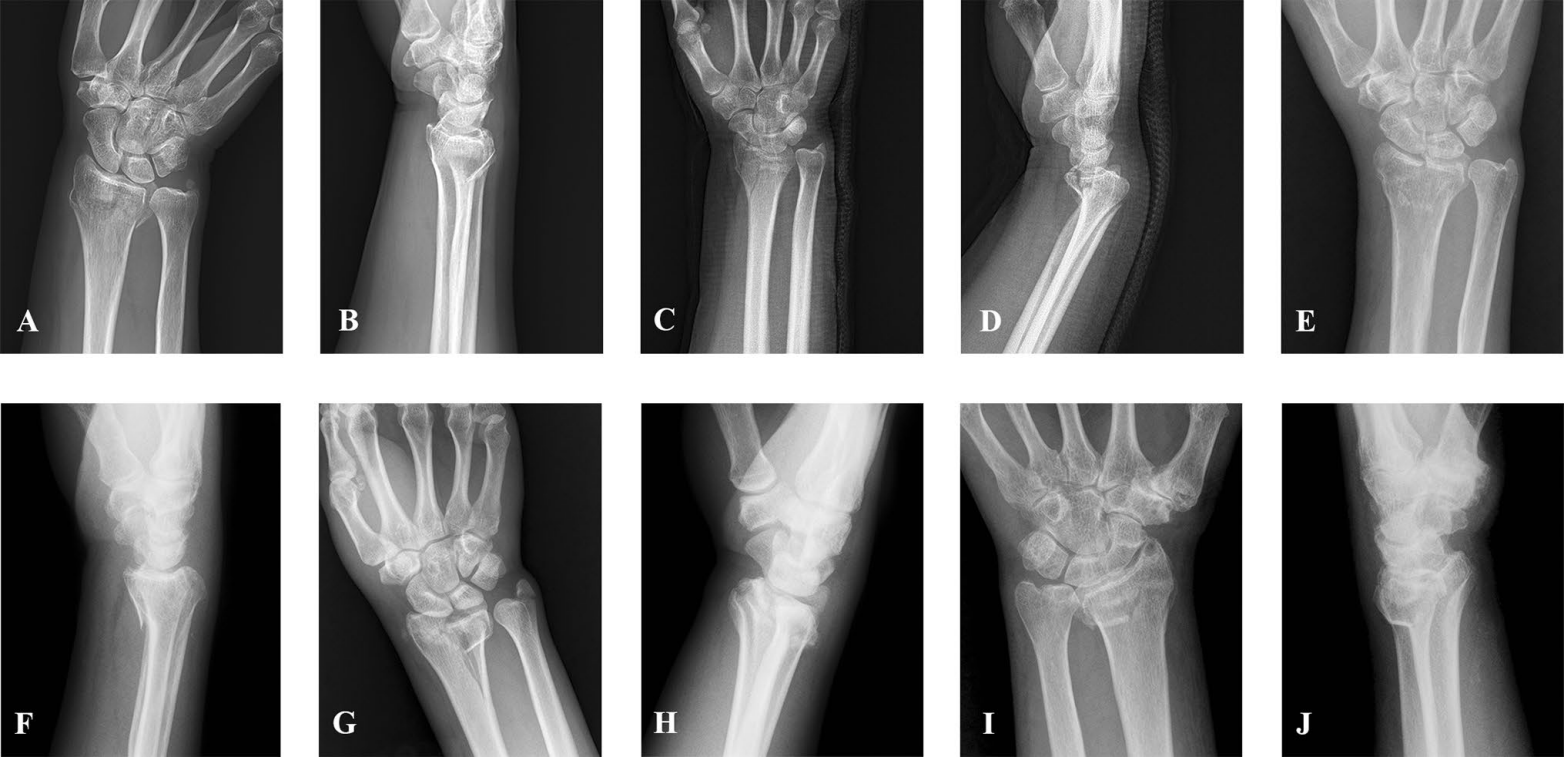
**a**, **b** show “┤” type fractures. **c, d** show “┤” type fractures. **e, f** show “┤” type fractures. **g, h** show “├” type fractures. **i, j** show “┼” type fractures. **a, c**, **e**, **g**, **i** the anteroposterior X-ray film; **b, d**, **f**, **h**, **j** represents the lateral X-ray film. Although fracture lines can be seen in (**a**, **c**), it is difficult to distinguish the distribution of fracture lines on the dorsal and dorsal sides. Although there are overlapping ulnar shadows in (**b**), dorsal displacement of the fracture block can be inferred. Similarly, the bone mass in (**d**) has a dorsal displacement. The sagittal fracture line can be clearly seen in (**e**, **g**) and the position of the fracture block can also be determined. However, it is difficult to obtain effective information in figure (**f**, **h**). The radius height shortening and fracture line distribution were observed in (**i**). Separation and displacement of the volar and dorsal fractures of the distal radius can be observed in **(J)**

By retrospectively analyzing the typical X-rays of each type before surgery, it was found that for the “┤” and “├” type fractures, the preoperative anteroposterior X-ray has a good indication. Because of the overlapping shadow of ulna, lateral X-ray is of great reference only when the free bone mass is obviously displaced. It is difficult to effectively observe the “┴” type fracture based on the anteroposterior X-ray, and the lateral X-ray may have a suggestive effect. The “┬” type fracture can be effectively observed by using the anteroposterior X-ray and the lateral X-ray. “┼” type fractures are generally accompanied by a large degree of displacement. Both the anteroposterior X-ray and the lateral X-ray can achieve effective observation. The main feature is the shortening of the humerus height (Fig. 7).

Due to the small sample size and the lack of surgical comparison, it is relatively difficult to add clinically relevant information to the existing manuscript. However, since the concept of surgery has been proposed, more appropriate cases could be enrolled and follow-up control studies would be performed in future.

The current study could help the surgeons to gain a deeper understanding of the distribution of the fracture lines in the intra-articular surface of the distal radius, which is significantly conducive to the surgical management of DRFs.

## Conclusion

In conclusion, mapping analyses of the number and distribution of the fracture lines could reveal the classification of distal radial intra-articular fractures. The results from the current study will be useful for the further understanding of the fracture patterns, providing the basis for the preoperative use of diagnostic imaging methods as well as for further developments in surgical approaches and fixation techniques for specific fracture types. However, the current study still contains a few notable limitations such as the small sample size and the assignment of all fracture fragments to one of the quadrants. Therefore, a large number of prospective studies are required in the future.
